# Self-aware cycle curriculum learning for multiple-choice reading comprehension

**DOI:** 10.7717/peerj-cs.1179

**Published:** 2022-12-05

**Authors:** Haihong Chen, Yufei Li

**Affiliations:** School of Mathematics and Computer Science, Chifeng University, Chifeng, China

**Keywords:** Machine reading comprehension, Multiple-choice, Self-aware, Cycle training strategy, Curriculum learning

## Abstract

Multiple-choice reading comprehension task has recently attracted significant interest. The task provides several options for each question and requires the machine to select one of them as the correct answer. Current approaches normally leverage a pre-training and then fine-tuning procedure that treats data equally, ignoring the difficulty of training examples. To solve this issue, curriculum learning (CL) has shown its effectiveness in improving the performance of models. However, previous methods have two problems with curriculum learning. First, most methods are rule-based, not flexible enough, and usually suitable for specific tasks, such as machine translation. Second, these methods arrange data from easy to hard or from hard to easy and overlook the fact that human beings usually learn from easy to difficult, and from difficult to easy when they make comprehension reading tasks. In this article, we propose a novel Self-Aware Cycle Curriculum Learning (SACCL) approach which can evaluate data difficulty from the model’s perspective and train the model with cycle training strategy. The experiments show that the proposed approach achieves better performance on the *C*^3^ dataset than the baseline, which verifies the effectiveness of SACCL.

## Introduction

Machine reading comprehension (MRC) (Min et al., 2020; Liu et al., 2018b; Peng et al., 2020; Yan et al., 2019; Peng et al., 2021; Nishida et al., 2019) is an important challenge in the field of natural language processing (NLP) and a basic task of textbased Question Answering, which is generally divided into four types (Liu et al., 2019a): cloze, multiple-choice, fragment extraction, and free generation. Among them, the multiple-choice MRC provides several options for each question, the machine needs to choose one of them as the correct answer, which has rich types such as commonsense reasoning and passage summarization. In addition, the answer may not appear in the original document, therefore the multiple-choice MRC is more challenging and requires a more in-depth understanding of the given document, question, and options.

Currently, the common practice for solving multiple-choice MRC tasks is to use pre-trained language models, and fine-tune them in a simple way. During training, all training examples are randomly processed, they are equal, which causes the model is not capable of learning the difficulty of the examples step by step. What’s more, the difficulty levels of training examples vary widely, some of which can be easily solved with simple word meaning, while others require complex inference. Table 1 shows an easy case and a hard case from the first free-form multiple-Choice Chinese machine reading Comprehension (*C*^*3*^) dataset (Sun et al., 2020). The easy case can be answered directly from the word “desert” that occurs in the document, and none of the words in the remaining three options appear in the document. In the hard case, all four options appear in the document, however, the question (*e.g*., luggage) is not explicitly presented. To answer this question, the machine needs to learn easy samples first, then learn hard samples, and learn step by step, instead of learning hard samples directly. In order to improve the performance of the task by arranging the data in a specific order rather than randomly, curriculum learning (CL) is considered in this article. It has been shown to improve the effectiveness of tasks (Wang, Chen & Zhu, 2021).

**Table 1:** An easy case and a hard case in *C*^3^ dataset.

Easy case	Hard case
男: 去沙漠旅游玩儿得怎么样?	女: 这次出差时间比较长,多带几件衣服.那边经常下雨,你得带把雨伞.
M: How about a trip to the desert?	F: This business trip is relatively long, so you could bring a few more clothes. It rains a lot over there, you have to bring an umbrella.
女: 挺有意思的,就是白天、晚上温差特别大,有点儿适应不了.	男: 都带了.帮我把水杯拿过来,上次就忘带了.
F: It’s very interesting, but the temperature difference between day and night is very large, and I can’t adapt to it.	M: All right. Bring me the tumbler, I forgot bringing it last time.
男: 夏天去的话容易晒伤皮肤.	女: 路上饿了怎么办?给你带些吃的东西吧.
M: In summer, it is easy to get sunburned.	F: What if you are hungry on the road? Let’s bring you something to eat.
女: 可不是嘛,我的脸到现在还疼呢.	男: 不用了,火车上有餐车,在那儿吃就行.
F: Well, my face still hurts.	M: No, there is a dining car on the train, just eat there.
Q: 女的去哪里玩儿了?	Q: 男的带的行李中不包括什么?
Question: Where did the girl go to play?	Question: What is not included in the man’s luggage?
A. 海边	A.衣服
A. Seaside	A. Clothes
B. 农村	B.食物
B. Village	B. Food
C. 沙漠	C.雨伞
C. Desert	C. Umbrella
D. 动物园	D.水杯
D. Zoo	D. Tumbler

The challenge of using CL lies in two aspects, difficulty assessment and training strategy. For the difficulty assessment, previous studies (Wang, Chen & Zhu, 2021) for evaluating the difficulty of text based on sentence length, the number of conjunctions (*e.g*., “but”, “while”), the number of phrases (*e.g*., verb phrases), the parse tree depth, word rarity, POS (part-of-speech) entropy, but these methods are mostly rule-based, which is only suitable for specific tasks and not flexible. For example, in machine translation, it is feasible to use rare words as a criterion for difficulty evaluation, but it is not feasible in multiple-choice reading comprehension, because the question may not be designed to rare words, that is, rare words are not keywords for answering questions. In addition, for the training strategy, previous CL approaches arrange data from easy to hard or from hard to easy. However, human beings usually learn from easy to difficult, and from difficult to easy in an iterative way when they make comprehension reading tasks.

In order to solve these problems, a novel Self-Aware Cycle Curriculum Learning (SACCL) approach is proposed to evaluate data difficulty from the model’s perspective and to train data with the cycle training strategy. Specifically, the **self-aware** approach can increase the flexibility and scalability of the model, which judges the difficulty of examples by the model itself. The cycle training strategy (CTS) approach iteratively trains the data, which can allow the model to reading comprehension in a way that is close to humans.

The contributions of our article are as follows:

1. In order to avoid the limitations of rule-based design, we propose a self-aware approach, which judges the difficulty of examples by the model itself.
2. In order to be more suitable for human thinking habits and fully train the model, we use the cycle training strategy to arrange the data.
3. We empirically show that our SACCL approach is effective, and achieve better performance than baseline on the multiple-choice Chinese machine reading comprehension (*C*^*3*^) dataset.

## Related work

### MRC datasets

Each type of MRC task has a more typical dataset. CNN & Daily Mail, proposed by Hermann et al. (2015), is a cloze-style reading comprehension dataset created from news articles using heuristics and is a classic dataset in the field of MRC. RACE is a multiple-choice dataset, and it covers a wide range of fields. It contains more than 100,000 questions posed by experts, and it focuses more on reasoning skills (Lai et al., 2017). SQuAD is a span extraction dataset proposed by Rajpurkar et al. (2016), which limits the answer to continuous fragments in the original text. It promoted the development of machine reading comprehension. MS MARCO is a free answering dataset (Nguyen et al., 2016). It does not limit the answer to a fragment in the document. It requires the machine to have the ability to comprehensively understand multi-document information and aggregate it to generate the answer to the question, which is closer to the real world.

Multiple-choice datasets provide a more accurate assessment of machine understanding of language, because questions and answers may come from human generalizations or summaries and may not appear directly in the document. Methods that rely only on information retrieval or word frequency cannot achieve good results. Sun et al. (2020) presents the first free-form multiple-choice Chinese machine reading comprehension dataset (*C*^*3*^). Various question types exist such as linguistic, domain-specific, arithmetic, connotation, implication, scenario, cause-effect, part-whole, and precondition in this dataset. Therefore it requires more advanced reading skills for the machine to perform well on this task. This article conducts related experiments on the *C*^*3*^ dataset.

### Multiple-choice methods

Previous research on multiple-choice is diverse. Dai, Fu & Yang (2021) propose an MRC model incorporating multi-granularity semantic reasoning. The model fuses the global information with the local multi-granularity information and uses it to make an answer selection. Sun et al. (2022) extract contextualized knowledge to improve machine reading comprehension. However, these approaches add extra features to the model, without the ease of in-depth analysis of the existing data. Zhang et al. (2020b) introduce the context vector of the syntax-guided to parse the passage and question separately, and obtain finer vector representations of passage and question, so as to give more accurate attention signals and reduce the influence of noise brought by long sentences. However, it is required to build a specific parsing tree for passages and questions. Zhang et al. (2020a) consider the interaction between documents, questions, and options, and introduce a gated fusion mechanism to filter out useless information, but the study does not consider the difficulty of the samples themselves. In this article, we propose the SACCL to mine the own characteristics of existing samples, and rationally arrange and use them to achieve better results.

### Curriculum learning

Extensive researches have been carried out, with the curriculum learning proposed in Bengio et al. (2009). It aims to facilitate the model training in a specific order, which leads to improved model performance (Hacohen & Weinshall, 2019; Xu et al., 2020). It has been applied to many fields, such as machine translation (Liu et al., 2020; Zhang et al., 2018; Platanios et al., 2019,), image recognition (Büyüktas, Erdem & Erdem, 2020; Huang et al., 2020), data-to-text generation (Chang, Yeh & Demberg, 2021), reinforcement learning (Narvekar et al., 2020), information retrieval (Penha & Hauff, 2020), speech emotion recognition (Lotfian & Busso, 2019), emotion recognition (Yang et al., 2022), spelling error correction (Gan, Xu & Zan, 2021). Curriculum learning has brought different degrees of improvement to these fields. Kumar et al. (2019) adopt CL to reinforcement learning to optimize the model parameters. CL also has shown to be useful for data processing to improve the quality of the training data (Huang & Du, 2019). Liu et al. (2018a) proposed a CL-NAG framework that utilizes curriculum learning to improve data utilization. CL-NAG makes full use of both noisy and low-quality corpora. In addition to the fact that the data is arranged in order from easiest to hardest, there are also some cases (Zhang et al., 2018, 2019; Kocmi & Bojar, 2017) where the data is arranged from difficult to easy with good results. With the wide application of deep learning in various fields, the use of CL to control the order of training data has received more and more attention.

## Method

In this section, we introduce our method in three parts. First, the task description describes input data and output data. Second, we construct a multiple-choice MRC model. Third, our SACCL approach includes difficulty assessment and training strategy.

### Task description

For multiple-choice reading comprehension, a document (denoted as *D*) and a question (denoted as *Q*), and a set of options (denoted as *O*) are given. Our task is to learn the predictive function *F*, which generates the answer (denoted as *A*) by receiving the document *D* and the related question *Q* and the options *O*, we define the task as follows:

(1) $$F(D,Q,O) = A \in ({o^1},{o^2},{o^3},{o^4})$$

The document $D = {d_1},{d_2}, \ldots ,{d_n}$ (*n* indicates the total number of words in the document), the question $Q = {q_1},{q_2}, \ldots ,{q_m}$ (*m* indicates the total number of words in the question), and the options $O = {o^1},{o^2},{o^3},{o^4},\,o_k^u = o_1^u,o_2^u,o_3^u,o_4^u, \ldots ,o_k^u$, where, $u = (1,2,3,4)$, each option consists of *K* words. *A* belongs to one of the four options, which is shown in Eq. (1). This setup challenges us to understand and reason about both the question and document in order to make an inference about the answer.

### Model

Following the implementation of BERT (Devlin et al., 2019), we concatenate the document, the question, and the option together as shown in Eq. (2), which is the input sequence. The input sequence is concatenated using two special tokens, < *CLS* > for the beginning, < *SEP* > for the end and the middle separator. The length of the input sequence is the sum of the document, the question, the option, and special tokens. We pass them to BERT and use a linear classifier on top of it to get the probability distribution. These are shown in Eqs. (3) and (4).

(2) $$Input = [ \lt CLS \gt D \lt SEP \gt Q \lt SEP \gt {o^i} \lt SEP \gt ]$$

(3) $${H = BERT\,(Input) }$$

(4) $${logits = Linear(H)}$$

where the logits are the probability distribution of the option.

### Our SACCL approach

Our SACCL (Self-Aware Cycle Curriculum Learning) approach consists of two important parts. Firstly, **Difficulty Assessment** judges the difficulty score of each sample. Secondly, **Training Strategy** arranges the order in which the samples are trained.

#### Difficulty assessment

**Figure 1:**
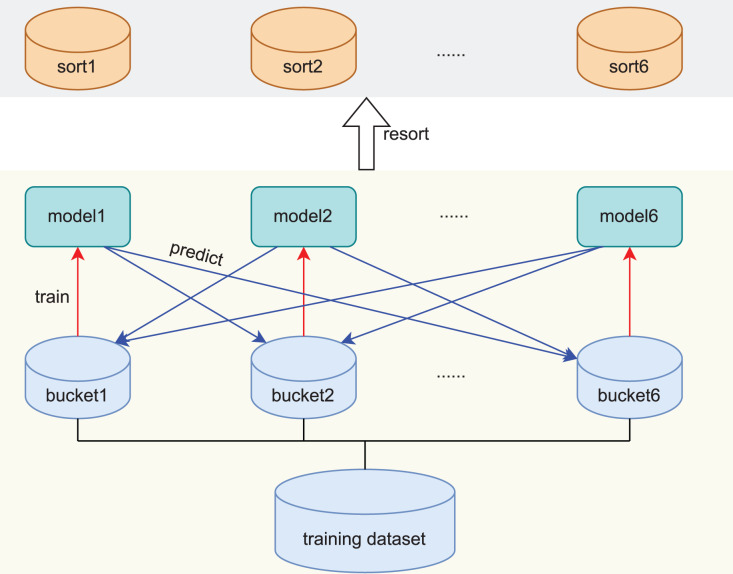
Creation and scoring process of the self-aware approach. The red arrow represents the training process and the blue arrow represents the prediction process.

As illustrated in Section Introduction, in order to avoid the limitations of rule-based design, and increase the scalability of the model, we design a **self-aware approach**, which independently judges the difficulty score of the samples by the model itself, and then sorts the samples according to the difficulty scores. The whole process consists of two parts. Firstly, the training model divides the original training data into six blocks, each block is trained with a model. Secondly, the trained models are used to score and sort the other samples, as shown in Fig. 1.

Let *R* be the training examples set, ${r_k}$ is the *k*-th example in *R*, difficulty assessment is to calculate the difficulty score ${s_k}$ of ${r_k}$ according to a certain evaluation standard, let *S* be the whole difficulty score set corresponding to *R*.

In order to evaluate the difficulty, we first randomly divide the training set *R* into six blocks according to the size of the dataset, denoted as $\big\{ {R_j^\prime \text{:} \,j = 1,2, \ldots ,6} \big\}$. Then we train six corresponding models $\left\{ {M_i^\prime \text{:} \,i = 1,2, \ldots ,6} \right\}$ on them, each model $M_i^\prime$ uses $R_j^\prime$ as the training set, and the remaining five pieces of data are used as the validation set to obtain the difficulty score. We also try to split the data into seven or five blocks, but the experiment works best when the data is divided into six blocks, so we choose to split the data into six blocks.

Specifically after training, $M_i^\prime$ predicts examples $R_j^\prime$, where $j \ne i$. *F* is the metric calculation formula, correct prediction is 1, error prediction is 0, as:

(5) $${s_{ki}} = F(M_i^\prime ,r_k^\prime )(r_k^\prime \in R_j^\prime ,j \ne i,i,j = 1,2, \ldots ,6)$$

The scores of each example are counted after all predictions are over, as:

(6) $${s_k} = \sum\limits_{i = 1}^5 {{s_{ki}}}$$

The examples are sorted according to ${s_k}$. If ${s_k} = 5$ indicates that all five models predict correctly. This type of example is the simplest and it is ranked first, and so on. If ${s_k} = 0$ indicates that all five models predict incorrectly. This type of example is the hardest and it is ranked last.

#### Training strategy

In order to train the data in an iterative manner, from easy to hard, and from hard to easy, which is more suitable for human thinking habits, we use the circular training strategy to arrange the data, which is called the cycle training strategy (CTS).

Our method arranges training examples *R* into cycle training strategy according to their difficulty scores *S* obtained in the previous section. After all samples are sorted according to *s*_*k*_, they are divided into *M* buckets: $M\,buckets\text{:} \,\left\{ {{B_t}\!\!:t = 1,2, \ldots ,M} \right\}$. In this article, we take *M* as 6. *B*_*1*_ is the easiest, and *B*_*6*_ is the hardest.

We design our training strategy in a multi-stage setting $\left\{ {{T_u}\!\!:u = 1,2, \ldots ,N} \right\}$. *N* indicates the count of epoch. In the first stage, only *B*_*1*_ is added for training. From the second stage, one *B*_*t*_ is added each time until all the data are added to the training set. After training an epoch, the last bucket added to the training set is subtracted. One bucket is subtracted from each round until only *B*_*1*_ is left. The data is shuﬄed for training at each stage. The progress of the training strategy is given in Algorithm 1.

**Algorithm 1:** Process of obtaining training dataset

**Input**: The training epoch count _epoch, the buckets count M, all the buckets B.
**Output: **The training dataset
1: *C* ⇐ [], *t* ⇐ 1, *increase* ⇐ *True*
2: **for** ${u} \in$ {0…_*epoch*} **do**
3: **if** increse **then**
4: *C* ⇐ *C* + *B_t_*
5: *t* ⇐ *t* + 1
6: **else**
7: *C* ⇐ *C* – *B_t_*
8: *t* ⇐ *t* − 1
9: **end if**
10: **if** *t* == *M* + 1 **then**
11: *increase* ⇐ *False*
12: *t* ⇐ M
13: **end if**
14: **if** (*t* == 1) **then**
15: *increase* ⇐ *True*
16: *t* ⇐ 2
17: **end if**
18: **end for**
19: return C

## Experiments

### Datasets

To verify the effectiveness of SACCL on multiple-choice tasks, the multiple-choice Chinese machine reading comprehension dataset (*C*^*3*^) is considered. This dataset contains 13,369 documents collected from questions in the general domain of the Chinese Proficiency Test and 19,577 multiple-choice free-form questions associated with these documents. In this dataset, there are 11,869 questions in the training set, 3,816 questions in the validation set, and 3,892 questions in the test set. The documents contain conversational form documents and non-dialogical documents with mixed topics (*e.g*., stories, news reports, monologues, or advertisements), which requires models to have stronger reasoning capabilities. *C*^*3*^tasks can be classified into *C*^*3*^-Dialogue $(C_D^3)$ and *C*^*3*^-Mixed $(C_M^3)$ based on the two types of documents. Also, 86.8% of the questions in this dataset require a combination of internal and external knowledge of the document (general world knowledge) to better understand the given text.

### Evaluation metric

Exact Match (EM), Precision, Recall, and macro-F1 (Rajpurkar et al., 2016) are used to evaluate the model performance. Exact Match measures the proportion of the correct results (including positive and negative cases) predicted by the model. A higher value of EM means that the model answers more questions correctly. Precision is the precision rate, indicating the proportion of the number of correctly predicted samples in all the samples whose prediction results are positive examples, and Recall is the recall rate, indicating the percentage of all the samples with positive results that are correctly predicted. F1 is used to measure the repetition rate of the prediction compared to the ground truth answer.

(7) $$Precision = \; \displaystyle{{TP} \over {TP + FP}}$$

(8) $$Recall = \; \displaystyle{{TP} \over {TP + FN}}$$

(9) $$macro - F1 = \; \displaystyle{{2 \cdot Precision \cdot Recall} \over {Precision + Recall}}$$

### Experimental settings

Our model is built using the PyTorch deep learning framework. We further pre-train with this model on one 3,090 GPU. We adjust the parameters in our model to what is shown in Table 2. In order to prevent model overfitting and excessive training time, the validation set is tested every round during the training phase of the model.

**Table 2:** Model parameter settings.

Parm type	Parm value
Batch size	24
Learning rate	1e−5
Epoch	20, 30
Max length	512
Dropout	0.15
Seed	66
Gradient accumulation steps	6
Warm-up proportion	0.1
Optimization function	Adam

### Experimental results

To evaluate the SACCL approach, the BERT (Devlin et al., 2019), RoBERTa (Liu et al., 2019b), and ALBERT (Lan et al., 2020) are used as the baseline. BERT is a bidirectional transformer (Vaswani et al., 2017) network. It uses the Encoder module in the transformer architecture and abandons the Decoder module, so that it automatically has bidirectional encoding capabilities and powerful feature extraction capabilities. BERT introduces masked language modeling (MLM) and next sentence prediction (NSP) tasks to train on plain text. RoBERTa is a finer-tuned version of the BERT model. The RoBERTa model removes the NSP task, uses a dynamic mask strategy, and trains with larger batch size and learning rate. ALBERT is a Lite BERT. It introduces parameter reduction technology, which significantly reduces the number of parameters of BERT without significantly compromising its performance, thereby improving parameter efficiency. The base pre-trained language models are used in the main experiments. We use BERT-base to re-implementation the article (Sun et al., 2020) and get better results on the test set.

#### Main comparison

In order to illustrate the effectiveness of our method, we first arrange the training data in the way of self-aware curriculum learning (SCL); that is, arranging the data from easy *B*_*1*_ to hard *B*_*6*_, and add a bucket each time, until all the data are added to the training set, and then train for several epochs until convergence. From Table 3, we can see that the SCL approach achieves 0.99% gain on EM over the results in the article (Sun et al., 2020) on *C*^*3*^-test. It can achieve improvements of 0.28% gain on EM over the baseline RoBERTa model on the *C*^*3*^-test, and achieve improvements of 1.92% gain on EM over the baseline ALBERT model on the *C*^*3*^-test, which proves that giving the model the easiest data at the beginning of training and then gradually increasing the difficulty of the data will get better results than randomly arranging the data.

**Table 3:** Comparison table of main experimental results.

	Dev				Test			
Method	EM	Prec	Rec	F1	EM	Prec	Rec	F1
								
BERT Base	65.70	–	–	–	64.70	–	–	–
BERT Base*	65.67	65.64	65.62	65.58	65.95	66.09	66.05	65.97
RoBERTa Base	67.76	67.67	67.77	67.70	66.98	66.94	66.97	66.91
ALBERT Base	53.87	53.68	53.86	53.68	53.62	53.48	53.66	53.51
BERT Base+SCL	65.85	65.80	65.85	65.78	65.69	65.74	65.78	65.66
RoBERTa Base+SCL	67.85	67.74	67.92	67.81	67.26	67.19	67.26	67.20
ALBERT Base+SCL	55.79	55.68	55.86	55.73	55.54	55.47	55.58	55.48
BERT Base+CTS	65.95	65.86	65.90	65.84	65.80	65.80	65.88	65.76
RoBERTa Base+CTS	68.18	68.07	68.23	68.13	67.54	67.50	67.57	67.51
ALBERT Base+CTS	55.79	55.68	55.86	55.73	55.54	55.47	55.58	55.48

**Note:**

* Our re-implementation.

**Figure 2:**
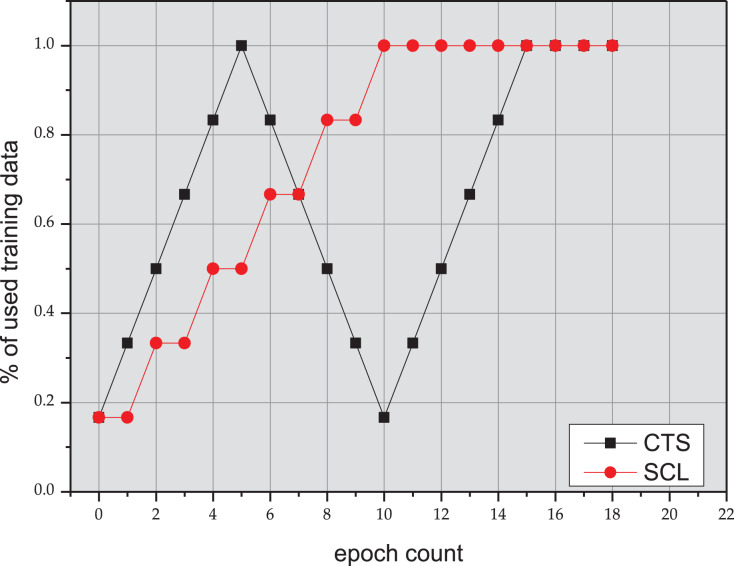
Training process of SCL and CTS.

In order to verify the effect of the CTS (Cycle Training Strategy) approach, we arrange the data in an iterative manner in Fig. 2. As shown in Table 3, the CTS approach achieves 1.1% gain on EM over the results in the article (Sun et al., 2020) on the *C*^*3*^-test. It can achieve improvements of 0.56% gain on EM over the baseline RoBERTa model on the *C*^*3*^-test, and achieve improvements of 1.92% gain on EM over the baseline ALBERT model on the *C*^*3*^-test, which proves that from easy to difficult, and then from difficult to easy, this repeated arrangement of data can get better results than SCL approach. In summary, the decent performance on the benchmark dataset can validate the effectiveness of the proposed CTS.

#### Comparison of self-aware approach and others

To better understand the SACCL approach, a comparison study of the self-aware approach and others is performed. First, the empirical-based approach evaluates sample difficulty based on human observation. This method takes into account the types of questions (Sun et al., 2020). Second, the document-length-based approach evaluates sample difficulty based on document length.

As for the empirical-based approach, we divide the training dataset into six buckets based on the types of questions. See Table A.1 for the types of questions. The results are shown in Table 4. CL and CTS experiments have not achieved better results than the self-aware approach. In the development set, the EM value of the self-aware method reaches 65.85, while the EM value of the empirical-based method is only 63.81. This indicates that the empirical-based approach does not work for all tasks. Because everyone’s knowledge is different, for the same question, people with relevant knowledge think the question is easy, and people without relevant knowledge think it is difficult, so this order may not really be from easy to difficult. We also counted the data distribution between each bucket and found that the data set divided by the self-aware approach is more balanced than the data set divided by empirical analysis, as shown in Table 5.

**Table 4:** The results of CL and CTS based on empirical analysis.

	Dev		Test	
Method	EM	F1	EM	F1
BERT base	65.70	–	64.70	–
BERT_empirical_CL	63.81	63.74	63.41	63.32
BERT_empirical_CTS	64.70	64.56	64.46	64.45

**Table 5:** The number of data in different buckets divided by three difficulty evaluation methods.

	Bucket1	Bucket2	Bucket3	Bucket4	Bucket5	Bucket6
Empirical based	1,762	346	898	5,035	1,105	2,723
Self-aware based	2,878	1,714	1,670	1,628	1,811	2,168
Document-length based	2,017	1,942	1,992	1,963	1,984	1,971

As for the second approach, we divide the training dataset into six buckets based on the document length. Bucket1 has the shortest data and bucket6 has the longest data. The results are shown in Table 6. The CL method is lower than the baseline on both the validation set and the test set, but the CTS method is higher than the baseline on both the validation set and the test set. The data distribution of this method is shown in Table 5. In both methods, the results of CTS are higher than CL, which can be proved the CTS approach effectiveness.

**Table 6:** The results of CL and CTS based on document length.

	Dev		Test	
Method	EM	F1	EM	F1
BERT base	65.70	–	64.70	–
BERT_doclen_CL	65.33	65.21	65.08	64.96
BERT_doclen_CTS	65.72	65.63	65.08	65.06

### Document length analysis

The maximum text length of the BERT model is 512. According to the analysis in Table 7, it is known that the ratio of *C*^*3*^ training dataset document length exceeding 512 is 14.2%. When we are dealing with multiple-choice tasks, the sum of the document length, question length, and option length exceeds 512, we select the document for additional processing. Because, generally speaking, the document length is the longest, or when the question length exceeds the document length, the total length will not exceed 512. Following (Sun et al., 2019), three strategies are chosen to truncate the document length. The first is deleted from the tail. The second is deleted from the head. And the third is deleted from the middle (we select the first 128 and the last 380 tokens). Through experimental analysis, it is found that most of the key information of long text comes from the head of the document in the *C*^*3*^ dataset, as shown in Table 8. Therefore the experiments are based on this setting in this article.

**Table 7:** C^3^ training dataset document length analysis.

	Average lengths	Max lengths	Exceeding ratio*	Samples
$C_d^3$	152	1,540	12.3%	5,856
$C_m^3$	290	1,274	15.9%	6,013
${C^3}$	222	1,540	14.2%	11,869

**Note:**

* The exceeding ratio means the percentage of the number of samples with a docment length exceeding 512.

**Table 8:** The results of truncating long document.

Method	Dev		Test	
	EM	F1	EM	F1
BERT base-middle	64.67	64.48	65.51	65.44
BERT base-head	64.04	63.92	65.18	65.07
BERT base-tail	65.67	65.58	65.95	65.97

## Conclusion

In this article, we present a Self-Aware Cycle Curriculum Learning (SACCL) approach for multiple-choice reading comprehension, which can judge the difficulty of the samples by the model itself, and learn in a loop like a human, that is from easy to hard and from hard to easy. The proposed SACCL is very effective and outperforms the baseline model. We also experiment with some other rule-based approaches and show interesting results, which demonstrate the effectiveness of our method. For future work, we consider transferring this method to other tasks such as machine translation, text classification, *etc*. to validate the robustness of our SACCL approach.

**Table A.1:** The type of question is ‘matching’.

Example 1
女: 今天是什么日子啊?加油站还排长队? F: What day is it today? Why are there long lines at gas stations?
男: 你没听说吗?明天汽油价格又要调整了,我昨天晚上就加满了. M: Haven’t you heard? The gas price will be changed again tomorrow. I filled it up last night.
Q: 汽油价格什么时候调整? Question: When will the price of petrol be adjusted?
A.下周 A. Next week
B.今天 B. Today
**C.明天C. Tomorrow**
D.后天D. The day after tomorrow

**Table A.2:** The type of question is ‘domain specific’.

Example 2
男: 你买了这么多东西啊!我来帮你拿吧. M: You bought so many things! Let me get that for you.
女: 谢谢你了.这些都是我马上要带回国的礼物,送给我的亲戚朋友们. F: Thank you very much. These are the gifts I will take back to my country soon for my relatives and friends.
男: 我来看看你要带些什么回去,哦,丝绸、扇子、象棋.哇,还有鞭炮!这东西不能带上飞机吧? M: Let me see what you want to take home. Oh, silk, fans, chess. Wow, and firecrackers! You can’t take this on the plane, can you?
女: 我知道,这是我买来准备今天晚上放的. F: I know. I bought it for tonight.
男: 你怎么想起来买这个? M: What made you want to buy this?
女: 过年的时候我看见你们放鞭炮,特别热闹,也特刺激.走前我也想试一试. F: I saw you set off firecrackers during the Spring Festival. It was very lively and exciting. I want to try it before I go.
男: 你不怕吗?很多女孩子都不敢放. M: Aren’t you afraid? A lot of girls are afraid to let go.
女: 我不怕,你们男孩子敢做的事我也敢做. F: I’m not afraid. I dare to do what you boys dare to do.
Q: 根据对话,可以知道什么? Question: What do we learn from the conversation?
**A.女的很勇敢A. The woman is brave.**
B.过年的时候女的放鞭炮了 B. The woman set off firecrackers during the Spring Festival.
C.男的今天晚上帮女的放鞭炮 C. The man tonight to help the woman set off firecrackers.
D.女的把礼物带回去放在自己家里 D. The woman took the gift home and kept it at her house.

**Table A.3:** The type of question is ‘domain specific’.

Example 3
男: 你这苹果怎么这么不新鲜啊. M: Your apples are so stale.
女: 这个季节哪还有什么新鲜的苹果啊,要吃新鲜的红富士得再等四五个月啊. F: There are no fresh apples in this season. You have to wait 4 or 5 months to eat fresh ‘Red Fuji’.
Q: 现在大概是什么季节? Question: What season is it now?
**A. 春天A. Spring**
B. 夏天 B. Summer
C. 秋天 C. Autumn
D. 冬天 D. Winter

## A Further explanation of empirical-based method

The empirical-based approach is entirely manual-based. In Table A.1, the type of question is ‘matching’, and the answer can be obtained from this sentence ‘The gas price will be changed again tomorrow’ in the text. This type of question is usually simple. In Table A.2, the question type is ‘linguistic’. This type of question usually requires understanding the whole document, which is more difficult than the ‘matching’ type. In Table A.3, the question type is ‘domain specific’. The question is: What season it is now? From the external knowledge, we can obtain that fresh ‘Red Fuji’ apples mature around September in China. And the correct answer is spring. Therefore, we argue that this type of question is usually more difficult than the ‘linguistic’ type. Bold in the options indicates the correct answer.

## Additional Information and Declarations

### Data Availability

The following information was supplied regarding data availability: The data and code are available at GitHub: https://github.com/nancychh12/SACCL; nancychh12. (2022). nancychh12/SACCL: first release (v1.0.0). Zenodo. https://doi.org/10.5281/zenodo.7118600.
